# Artificial intelligence prediction of therapy response in newly diagnosed adult severe primary immune thrombocytopenia

**DOI:** 10.1016/j.rpth.2026.106916

**Published:** 2026-09-16

**Authors:** Xiaolin Zhai, Saibing Qi, Xiyan Wang, Wei Zhang, Mengyun Chen, Qiujin Shen, Xiaowen Gong, Wangsong Zhai, Sichang Liu, Xuan Liang, Yueshen Ma, Zhen Song, Robert Peter Gale, Juan Wang, Renchi Yang, Xiaofan Liu, Lei Zhang, Junren Chen

**Affiliations:** 1 State Key Laboratory of Experimental Hematology, National Clinical Research Center for Blood Diseases, Haihe Laboratory of Cell Ecosystem, Institute of Hematology & Blood Diseases Hospital, Chinese Academy of Medical Sciences & Peking Union Medical College, Tianjin, China; 2 Tianjin Institutes of Health Science, Tianjin, China; 3 Department of Hematology, Cangzhou Central Hospital, Hebei, China; 4 Yidu Cloud Technology Inc, Beijing, China; 5 Centre for Haematology, Department of Immunology and Inflammation, Imperial College of Science, Technology and Medicine, London, UK

**Keywords:** artificial intelligence, complete blood count, immune thrombocytopenia, prediction, response

## Abstract

**Background:**

Newly diagnosed severe primary immune thrombocytopenia (ITP) with a platelet count of ≤10 × 10^9^/L and clinically important bleeding requiring treatment is a medical emergency.

**Objectives:**

Using early kinetics of complete blood counts (CBC) to predict time interval to confirmed response in newly diagnosed severe primary ITP in adults.

**Methods:**

We used a training cohort of 85 adults with newly diagnosed severe primary ITP to develop an artificial intelligence model predicting confirmed response. The model used CBC values over the initial 5 days of therapy to classify subjects into 3 strata: high-confidence fast response, lower-confidence fast response, and slow/no response. We validated the model in an independent cohort of 59 subjects.

**Results:**

Concordance between the model-computed risk stratification and time to confirmed response is 0.78 (95% CI, 0.73, 0.82) and 0.78 (95% CI, 0.72, 0.83) in the training and validation cohorts, respectively. SHapley Additive exPlanations analyses indicated that the most informative covariates are age and kinetics of platelet count, mean red blood cell (RBC) volume, immature platelet fraction, standard deviation of RBC distribution width, mean RBC hemoglobin concentration, and immature reticulocyte percentage during days 3-5 of therapy.

**Conclusion:**

Our study should be treated as hypothesis-generating. Early dynamic patterns of CBC values might be used by artificial intelligence to predict the likelihood and speed of reaching confirmed response in adults with newly diagnosed severe primary ITP and might assist decision on timely therapy adjustment. Prospective testing and independent validation in non-Chinese cohorts and cohorts treated with different paradigms are needed.

## Essentials

• Treatment efficacy in newly diagnosed severe primary immune thrombocytopenia is unpredictable.
• We develop a predictive artificial intelligence model monitoring early dynamic patterns of complete blood counts.
• The model is developed using 85 subjects and validated in an independent cohort of 59 subjects.
• Our study suggests the hypothesis that early stratification might inform therapy adjustments.

## 1 Introduction

Primary immune thrombocytopenia (ITP) is an acquired autoimmune disorder characterized by increased destruction of platelets and a low platelet count in the absence of other causes or disorders associated with thrombocytopenia [1]. Initial therapy of newly diagnosed ITP focuses on glucocorticoid, administered alone or in combination with additional medication [[1], [2], [3], [4], [5], [6], [7], [8]]. There are several new therapies for initial or repeated therapy failures [9,10].

Newly diagnosed primary ITP with a platelet count of ≤10 × 10^9^/L and clinically important bleeding requiring treatment is a severe medical emergency [11,12]. There is an urgent need to restore normal thrombopoiesis and reverse thrombocytopenia using glucocorticoid, intravenous immunoglobulin (IVIG), thrombopoietin receptor agonist (TPO-RA), vinca alkaloid, and/or other drug options [3]. However, there is no widely accepted method for predicting which patients would respond to initial therapy. Some studies have reported covariates associated with the likelihood of response to therapy in persons with ITP, but not in persons with newly diagnosed severe primary ITP [[13], [14], [15], [16], [17], [18]].

Recent data indicate that even complete blood counts (CBC)—the most basic medical test—contain hitherto-untapped valuable information [19]. We hypothesized that artificial intelligence (AI) might uncover information embedded in dynamic CBC data that can serve as an early indicator of responsiveness to therapy. We used data from 85 consecutive adults with newly diagnosed severe primary ITP and dynamic aGVHD Onset Anticipation Tianjin (daGOAT)—a machine learning algorithm which we devised previously—to develop a model for predicting treatment response [20,21]. We found CBC signatures predictive of early therapy response and validated the model in an independent dataset of 59 subjects.

## 2 Methods

### 2.1 Definitions

We defined “newly diagnosed” as persons diagnosed ≤3 months. “Severe” was defined as ITP with a “baseline” platelet count (defined as the lowest value ≤2 days before therapy start) of ≤10 × 10^9^/L and ≥grade 3 cutaneous bleeding or ≥grade 2 mucosal or internal bleeding according to the ITP-specific bleeding assessment tool [1,3,11,12,22]. The bleeding score based on the modified World Health Organization (WHO) bleeding scale was also evaluated for each patient [23]. “Day 0” was defined as the day of starting therapy after hospital admission. “Confirmed response” was defined as a platelet count ≥30 × 10^9^/L for ≥7 days [11]. “Time to confirmed response” was the interval from day 0 to the first of 7 consecutive days of platelet count ≥30 × 10^9^/L.

### 2.2 Subjects

The training cohort was subjects >18 years old with newly diagnosed severe primary ITP receiving glucocorticoid(s) alone or in combination with ≥1 other drug as initial therapy at the Institute of Hematology, Chinese Academy of Medical Sciences (IHCAMS [Tianjin, China]) during January 1, 2012, to July 31, 2021. The validation cohort was subjects receiving glucocorticoid(s) alone or in combination with ≥1 other drug as initial therapy at the Cangzhou Central Hospital (CCH [Hebei, China]) during October 1, 2010, to July 31, 2025. The Ethics Committee at the IHCAMS approved the study (IIT2021049-EC-2) and waived requirement for written informed consent in compliance with precepts of the Declaration of Helsinki.

### 2.3 Data preprocessing

For all the subjects in the training and validation cohorts, we applied “time-limited sample-and-hold” [20] to all the CBC covariates: a measured value was assumed to be valid for a “grace period” of 3 additional days after measurement, unless a new measurement was made during the grace period. If a new CBC test was not performed in time, values were treated as missing, starting from the end of the grace period until the next measurement. The daGOAT algorithm permits missing data (*vide infra*).

### 2.4 AI model development

We constructed an AI model to forecast the likelihood of achieving confirmed response in adults newly diagnosed with severe primary ITP. Input covariates were sex, age, and daily values (from day 1 to day 5) of 20 CBC covariates including 2 covariates related to platelets (platelet count and immature platelet fraction [IPF]), 7 related to white blood cells (white blood cell, neutrophil, eosinophil, basophil, monocyte, lymphocyte, and immature granulocyte counts), and 11 related to red blood cells (red blood cell, reticulocyte and nucleated red blood cell counts, hemoglobin, hematocrit, mean corpuscular volume [MCV], mean corpuscular hemoglobin, mean corpuscular hemoglobin concentration [MCHC], immature reticulocyte fraction [IRF], reticulocyte hemoglobin equivalent, and standard deviation of red cell distribution width [RDW-SD]). Platelet distribution width was excluded because measurements were not available for all the training-cohort subjects during the initial 5 days of therapy.

We modeled the daily likelihood score ϕ(t) for achieving confirmed response as

$$\phi \left(t\right)=\rho \left(z\right)+\sum_{k=1}^{20}I_{k\tau }\theta_{k}\left(x_{k}\left(t\right),t\right)\text{,}$$

where z is a 2-variable vector, $\left(\text{age,}\;\text{sex}\right)$; $x_{k}\left(t\right)$ is the kth CBC covariate’s value on day t; $I_{kt}=0$ if the value of $x_{k}$ is unavailable on day t, and $I_{k\tau }=1$ otherwise; and ρ(z) and $\theta_{k}\left(x_{k}\left(t\right),t\right)$ are contributions of z and $x_{k}\left(t\right)$, respectively, to the likelihood score ϕ(t). To accommodate potential nonlinearity, we did not presume any mathematical form for $\theta_{k}\left(x,t\right)$. Rather, we only assumed that $\theta_{k}\left(x,t\right)$ is “smooth” with respect to t; that is, *a priori*, the value of $\theta_{k}\left(x,t\right)$ does not have rapid, drastic up-and-down swings between neighboring days unless data strongly suggest otherwise. Under these conditions—that is, when the physiological process underlying early therapy response is multidimensional but smooth—the daGOAT algorithm outperforms other benchmark algorithms in dynamic updating of risk scores [20].

### 2.5 Trinary classification of subjects

We classified subjects based on the speed and magnitude of initial platelet count increase into “fast response” (≥30 × 10^9^/L in ≥1 test within 5 days after therapy start) and “slow/no response” (failing to meet the criteria for “fast response”). Next, we used the daGOAT algorithm–trained model to further classify the fast-response subjects into “high-confidence fast response” and “lower-confidence fast response,” as follows: fast-response subjects whose model-computed likelihood score for achieving confirmed response was equal to or larger than the median value of the subjects in the training cohort on ≥4 days during days 1 to 5 and on each day during days 3 to 5 were classified as high-confidence fast response, whereas the other fast-response subjects were classified as lower-confidence fast response.

### 2.6 Online calculator

The proposed model for trinary classification of subjects that was fitted using data from the 85 subjects at the IHCAMS is made publicly accessible as an online calculator (version 1.0) at https://itp-calculator.shinyapps.io/shiny_itp/, with the target user population being researchers interested in conducting validation analyses of the model or using the model as a risk stratification aid for targeted recruitment of subjects at risk for delayed response into new therapy trials.

### 2.7 Statistics

ITP-targeting drugs were classified into 7 types: glucocorticoids, other steroids (eg, danazol), IVIG, recombinant human thrombopoietin (rhTPO), TPO-RA, cyclosporine A, and vinca alkaloids.

All analyses were conducted using the R computing language (www.r-project.org; version 4.4.1). Hazard ratios (HRs), concordance scores, and the Cox model were computed using the R function “coxph” in the “survival” package (version 3.6.4). Comparison of concordance scores was computed using the R function “compareC” in the “compareC” package (version 1.3.3).

For each CBC covariate, for each day from day 1 to day 5, and for each subject in the training cohort, we calculated the corresponding SHapley Additive exPlanations (SHAP) value—setting model outputs corresponding to predicting high-confidence fast response, predicting lower-confidence fast response, and predicting slow/no response as 2/3, 1/3, and 0, respectively—using the R function “explain” in the “fastshap” package (version 0.1.1). To assess the relative overall importance of the CBC covariates, for each covariate, we calculated the average absolute value of the sum of SHAP values over days 1 to 5 in the training cohort (average was taken across all the subjects in the training cohort). Furthermore, to assess relative daily contributions of the CBC covariates, we calculated the average absolute SHAP value of each CBC covariate on each day after therapy start in the training cohort (again, average was taken across all the training-cohort subjects).

All reported *P* values are 2-sided without adjustment for multiple-hypothesis testing. Statistical significance was defined as unadjusted, 2-sided *P* < .05.

## 3 Results

### 3.1 Baseline covariates in the training cohort

There were 85 consecutive adults with newly diagnosed severe primary ITP receiving glucocorticoid(s) in initial therapy at the IHCAMS from January 1, 2012, to July 31, 2021. Before admission to the IHCAMS, 21 subjects had previously received glucocorticoid(s) and 1 had received a combination of IVIG (4 days), rhTPO (2 days), and platelet transfusion (1 unit). Baseline covariates at admission are displayed in Table 1. The median baseline platelet count was 3 × 10^9^/L (IQR, 2-7 × 10^9^/L). Sixty-nine (81%), 48 (56%), and 22 (26%) subjects had ≥grade 3 cutaneous bleeding, ≥grade 2 mucosal bleeding, and ≥grade 2 internal bleeding, respectively. Initial glucocorticoid therapy at the IHCAMS consisted of intravenous methylprednisolone (*n* = 80; median cumulative dose = 200 mg [IQR, 200-200 mg] during days 1-5), oral prednisolone (*n* = 3; median = 200 mg [IQR, 36-200 mg]), and other regimens (Table 1). Most (*n* = 66 [78%]) subjects received ≥1 other drug in addition to glucocorticoid(s) during days 1 to 5 (Table 1). The most common add-on medications were IVIG (*n* = 31 [47% of 66]), rhTPO (*n* = 13 [20%]), and “IVIG + rhTPO” (*n* = 11 [17%]).

**Table 1:** Baseline characteristics of patients.

Clinical covariates and medication	Training cohort (*n* = 85)	Validation cohort (*n* = 59)	*P* value
Age (y), median (range)	46 (19-75)	50 (21-76)	.046^a^
Sex, *n* (%)			.78^b^
Male	32 (38)	20 (34)	
Female	53 (62)	39 (66)	
Race and ethnicity, *n* (%)			>.99^b^
East Asian	85 (100)	59 (100)	
Baseline platelet count (10^9^/L), median (IQR)	3 (2-7)	3 (1-5)	.03^a^
WHO bleeding scale, *n* (%)			.047^b^
2	71 (84)	57 (97)	
3	13 (15)	2 (3)	
4	1 (1)	0 (0)	
Skin bleeding scale according to ITP-BAT, *n* (%)			.42^b^
0	4 (5)	6 (10)	
1	0 (0)	0 (0)	
2	12 (14)	9 (15)	
3	69 (81)	44 (75)	
4	0 (0)	0 (0)	
Mucosal bleeding scale according to ITP-BAT, *n* (%)			<.001^b^
0	28 (33)	18 (31)	
1	9 (11)	16 (27)	
2	13 (15)	25 (42)	
3	33 (39)	0 (0)	
4	2 (2)	0 (0)	
Organ and internal mucosal bleeding scale according to ITP-BAT, *n* (%)			.001^b^
0	39 (46)	38 (64)	
1	24 (28)	8 (14)	
2	6 (7)	11 (19)	
3	4 (5)	2 (3)	
4	12 (14)	0 (0)	
Clinically relevant bleeding, *n* (%)	85 (100)	59 (100)	>.99^b^
Anti–glycoprotein Ib/IX antibody, *n* (%)^c^			NA
Positive	5 (6)	–	
Negative	73 (94)	–	
Anti–glycoprotein Ia/IIa antibody, *n* (%)^c^			NA
Positive	10 (13)	–	
Negative	68 (87)	–	
Anti–glycoprotein IIb/IIIa antibody, *n* (%)^c^			NA
Positive	31 (40)	–	
Negative	47 (60)	–	
Anti–β2 glycoprotein I antibody IgA, *n* (%)^d^			NA
Positive	0 (0)	–	
Negative	59 (100)	–	
Anti–β2 glycoprotein I antibody IgG, *n* (%)^d^			NA
Positive	8 (14)	–	
Negative	51 (86)	–	
Anti–β2 glycoprotein I antibody IgM, *n* (%)^d^			NA
Positive	2 (3)	–	
Negative	57 (97)	–	
Lymphocyte/monocyte ratio, median (range)	4 (1-248)	4 (0-113)	.14^a^
Hemoglobin (g/dL), median (IQR)	13 (12-15)	12 (11-14)	.14^a^
Direct Coombs test, *n* (%)^e^			NA
Positive	5 (7)	–	
Negative	63 (93)	–	
Had received treatment before admission to IHCAMS or CCH, *n* (%)			<.001^b^
Glucocorticoid	21 (25)	0 (0)	
IVIG + rhTPO + platelet transfusion	1 (1)	0 (0)	
None	63 (74)	59 (100)	
Type of glucocorticoid during days 1-5, *n* (%)			.004^b^
Methylprednisolone	80 (94)	49 (83)	
Prednisone	3 (4)	0 (0)	
Dexamethasone	1 (1)	3 (5)	
Methylprednisolone + prednisolone	1 (1)	0 (0)	
Methylprednisolone + dexamethasone	0 (0)	7 (12)	
Additional medication during days 1-5, *n* (%)			.04^b^
None	19 (22)	26 (44)	
IVIG	31 (36)	16 (27)	
rhTPO	13 (15)	14 (24)	
Danazol	2 (2)	0 (0)	
IVIG + rhTPO	11 (13)	3 (5)	
IVIG + eltrombopag	2 (2)	0 (0)	
IVIG + danazol	3 (4)	0 (0)	
rhTPO + danazol	2 (2)	0 (0)	
Eltrombopag + cyclosporine A	1 (1)	0 (0)	
IVIG + danazol + vindesine	1 (1)	0 (0)	

CCH, Cangzhou Central Hospital; IHCAMS, Institute of Hematology, Chinese Academy of Medical Sciences; ITP-BAT, immune thrombocytopenia–specific bleeding assessment tool; IVIG, intravenous immunoglobulin; NA, not applicable; rhTPO, recombinant human thrombopoietin; WHO, World Health Organization.

a Wilcoxon test.

b χ^2^ test.

c Baseline serum levels of anti–glycoprotein Ib/IX antibody, anti–glycoprotein Ia/IIa antibody, and anti–glycoprotein IIb/IIIa antibody are unavailable in 7 subjects in the training cohort and all 59 subjects in the validation cohort.

d Baseline serum levels of anti–β2 glycoprotein I antibodies IgA, IgG, and IgM are unavailable in 26 subjects in the training cohort and all 59 subjects in the validation cohort.

e Direct Coombs test results are unavailable in 17 subjects in the training cohort.

Of the 85 subjects in the training cohort, 47 (55%), 63 (74%), 78 (92%), and 81 (95%) achieved confirmed response by days 5, 10, 20, and 30, respectively. Concordance between the baseline platelet count and interval to confirmed response was only 0.52 (95% CI, 0.44, 0.60). Concordance between WHO bleeding score and interval to confirmed response was 0.53 (95% CI, 0.48, 0.58).

### 3.2 Taking into account the dynamics of CBC covariates

**Figure 1:**
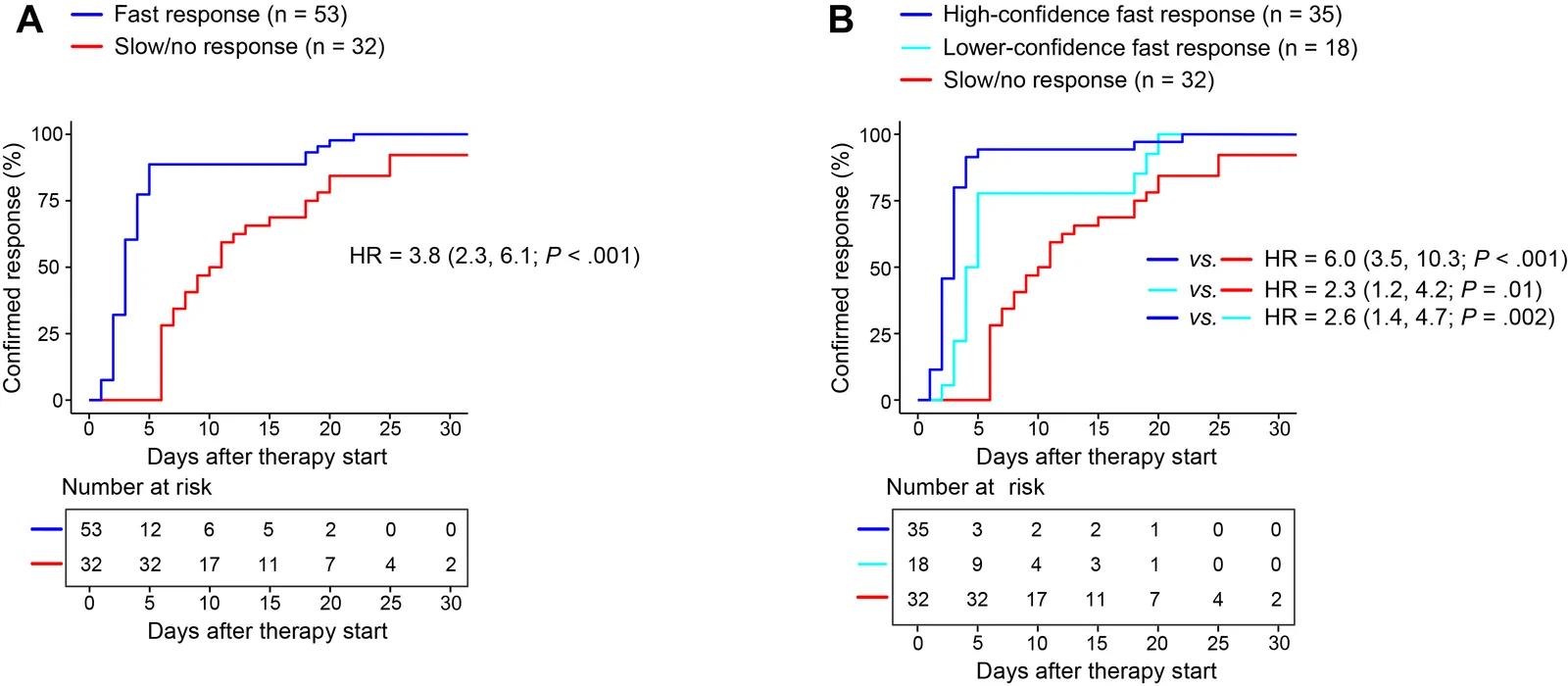
Cumulative incidence of confirmed response in the training cohort. (A) ‘Fast response’ vs ‘slow/no response’. (B) ‘High-confidence fast response’ vs ‘lower-confidence fast response’ vs ‘slow/no response’.

Two (2%), 3 (4%), 30 (35%), and 50 (59%) training-cohort subjects had 0, 1, 2, and ≥3 CBC measurements during days 1 to 5, respectively. Fifty-three (62%) subjects had “fast response,” that is, their platelet count was ≥30 × 10^9^/L in ≥1 test during days 1 to 5. The other 32 (38%) subjects were classified as slow/no response, including 2 who did not undergo the CBC test during days 1 to 5. The initial fast increase of platelet count was associated with a briefer interval to confirmed response (concordance score = 0.72 [95% CI, 0.67, 0.76]; Figure 1A).

Using a daGOAT algorithm–trained model, we further classified the fast response subjects into 2 strata, high-confidence fast response (*n* = 35; 41%) and lower-confidence fast response (*n* = 18; 21%). Figure 1B displays the 3 final strata’s cumulative incidence functions of confirmed response. Concordance between the daGOAT model–defined trinary classification and time to confirmed response was 0.78 (95% CI, 0.73, 0.82), significantly better than considering only the platelet count (*P* < .001). HR for reaching confirmed response was 6.0 (95% CI, 3.5, 10.3; *P* < .001) and 2.3 (95% CI, 1.2, 4.2; *P* = .01) in the high- and lower-confidence fast response strata, respectively, compared with the slow/no response stratum.

### 3.3 Baseline differences among the 3 strata in the training cohort

There was no significant difference in sex, age, bleeding scores, or the baseline values of platelet count, antiglycoprotein antibodies, or lymphocyte/monocyte ratio among the 3 strata in the training cohort (Table 2). There was also no difference in the frequency of IVIG, TPO-RA (eltrombopag), danazol, cyclosporine A, or vinca alkaloid (vindesine) use during days 1 to 5 among the 3 strata. A smaller proportion of the high-confidence fast response subjects received rhTPO before (and including) day 5 compared with the subjects in the other 2 strata (6 of 35 vs 20 of 50; *P* = .04; Table 2). However, concordance between rhTPO use and time to confirmed response was only 0.56 (95% CI, 0.50, 0.61).

**Table 2:** Baseline difference among the 3 strata in the training and validation cohorts.

Clinical covariates and medication	Training cohort				Validation cohort			
	High-confidence fast response (*n* = 35)	Lower-confidence fast response (*n* = 18)	Slow/no response (*n* = 32)	*P* value	High-confidence fast response (*n* = 12)	Lower-confidence fast response (*n* = 27)	Slow/no response (*n* = 20)	*P* value
Age (y), median (range)	51 (21-75)	45 (20-60)	46 (19-68)	.42^a^	52 (22-72)	50 (21-75)	41 (22-76)	.62^a^
Sex, *n* (%)				.66^b^				.048^b^
Male	12 (34)	6 (33)	14 (44)		1 (8)	13 (48)	6 (30)	
Female	23 (66)	12 (67)	18 (56)		11 (92)	14 (52)	14 (70)	
Baseline platelet count (10^9^/L), median (IQR)	3 (2-6.5)	3 (2-8)	3.5 (2-5.5)	.91^a^	2 (2-4)	3 (1-5)	2.5 (2-4)	>.99^a^
WHO bleeding scale, *n* (%)				.055^b^				.75^b^
2	34 (97)	13 (72)	24 (75)		12 (100)	26 (96)	19 (95)	
3	1 (3)	5 (28)	7 (22)		0 (0)	1 (4)	1 (5)	
4	0 (0)	0 (0)	1 (3)		0 (0)	0 (0)	0 (0)	
Skin bleeding score according to ITP-BAT, *n* (%)				.77^b^				.22^b^
0	1 (3)	1 (6)	2 (6)		0 (0)	3 (11)	3 (15)	
1	0 (0)	0 (0)	0 (0)		0 (0)	0 (0)	0 (0)	
2	4 (11)	4 (22)	4 (13)		4 (33)	2 (7)	3 (15)	
3	30 (86)	13 (72)	26 (81)		8 (67)	22 (81)	14 (70)	
4	0 (0)	0 (0)	0 (0)		0 (0)	0 (0)	0 (0)	
Mucosal bleeding score according to ITP-BAT, *n* (%)				.49^b^				.51^b^
0	15 (43)	4 (22)	9 (28)		3 (25)	11 (41)	4 (20)	
1	3 (9)	3 (17)	3 (9)		4 (33)	7 (26)	5 (25)	
2	6 (17)	4 (22)	3 (9)		5 (42)	9 (33)	11 (55)	
3	11 (31)	6 (33)	16 (50)		0 (0)	0 (0)	0 (0)	
4	0 (0)	1 (6)	1 (3)		0 (0)	0 (0)	0 (0)	
Organ and internal mucosal bleeding score according to ITP-BAT, *n* (%)				.33^b^				.98^b^
0	17 (49)	7 (39)	15 (47)		8 (67)	17 (63)	13 (65)	
1	12 (34)	5 (28)	7 (22)		2 (17)	3 (11)	3 (15)	
2	2 (6)	2 (11)	2 (6)		2 (17)	6 (22)	3 (15)	
3	3 (9)	0 (0)	1 (3)		0 (0)	1 (4)	1 (5)	
4	1 (3)	4 (22)	7 (22)		0 (0)	0 (0)	0 (0)	
Anti–glycoprotein Ib/IX antibody, *n* (%)^c^				.16^b^				NA
Positive	4 (12)	1 (6)	0 (0)		–	–	–	
Negative	29 (88)	16 (94)	28 (100)		–	–	–	
Anti–glycoprotein Ia/IIa antibody, *n* (%)^c^				.87^b^				NA
Positive	5 (15)	2 (12)	3 (11)		–	–	–	
Negative	28 (85)	15 (88)	25 (89)		–	–	–	
Anti–glycoprotein IIb/IIIa antibody, *n* (%)^c^				.99^b^				NA
Positive	13 (39)	7 (41)	11 (39)		–	–	–	
Negative	20 (61)	10 (59)	17 (61)		–	–	–	
Anti–β2 glycoprotein I antibody IgA, *n* (%)^d^				>.99^b^				NA
Positive	0 (0)	0 (0)	0 (0)		–	–	–	
Negative	27 (100)	10 (100)	22 (100)		–	–	–	
Anti–β2 glycoprotein I antibody IgG, *n* (%)^d^				.59^b^				NA
Positive	5 (19)	1 (10)	2 (9)		–	–	–	
Negative	22 (81)	9 (90)	20 (91)		–	–	–	
Anti–β2 glycoprotein I antibody IgM, *n* (%)^d^				.80^b^				NA
Positive	1 (4)	0 (0)	1 (5)		–	–	–	
Negative	26 (96)	10 (100)	21 (95)		–	–	–	
Lymphocyte/monocyte ratio, median (range)	4 (2-10)	4 (2-248)	4 (1-9)	.44^a^	4 (1-8)	4 (1-113)	5 (0-28)	.42^a^
Had received treatment before admission to IHCAMS or CCH, *n* (%)				.11^b^				>.99^b^
Glucocorticoid	5 (14)	6 (33)	10 (31)		0 (0)	0 (0)	0 (0)	
IVIG + rhTPO + platelet transfusion	0 (0)	1 (6)	0 (0)		0 (0)	0 (0)	0 (0)	
None	30 (86)	11 (61)	22 (69)		12 (100)	27 (100)	20 (100)	
Medication during days 1-5, *n* (%)								
Glucocorticoid	35 (100)	18 (100)	32 (100)	>.99^b^	12 (100)	27 (100)	20 (100)	>.99^b^
IVIG	25 (71)	9 (50)	14 (44)	.06^b^	2 (17)	10 (37)	7 (35)	.43^b^
Eltrombopag	1 (3)	1 (6)	1 (3)	.87^b^	0 (0)	0 (0)	0 (0)	>.99^b^
rhTPO	6 (17)	4 (22)	16 (50)	.01^b^	4 (33)	5 (19)	8 (40)	.25^b^
Danazol	2 (6)	2 (11)	4 (13)	.61^b^	0 (0)	0 (0)	0 (0)	>.99^b^
Cyclosporine A	0 (0)	1 (6)	0 (0)	.15^b^	0 (0)	0 (0)	0 (0)	>.99^b^
Vindesine	0 (0)	1 (6)	0 (0)	.15^b^	0 (0)	0 (0)	0 (0)	>.99^b^
Addition of drug(s) between day 6 and confirmed response								
IVIG	0 (0)	1 (6)	6 (19)	.02^b^	0 (0)	0 (0)	1 (5)	.37^b^
Eltrombopag	0 (0)	0 (0)	2 (6)	.18^b^	0 (0)	0 (0)	1 (5)	.37^b^
rhTPO	2 (6)	1 (6)	4 (13)	.54^b^	0 (0)	2 (7)	0 (0)	.29^b^
Danazol	0 (0)	1 (6)	5 (16)	.04^b^	0 (0)	0 (0)	0 (0)	>.99^b^
Cyclosporine A	0 (0)	0 (0)	1 (3)	.43^b^	0 (0)	1 (4)	0 (0)	.55^b^
Vindesine	0 (0)	0 (0)	2 (6)	.18^b^	0 (0)	0 (0)	0 (0)	>.99^b^

CCH, Cangzhou Central Hospital; IHCAMS, Institute of Hematology, Chinese Academy of Medical Sciences; ITP-BAT, immune thrombocytopenia–specific bleeding assessment tool; IVIG, intravenous immunoglobulin; NA, not applicable; rhTPO, recombinant human thrombopoietin; WHO, World Health Organization.

a Kruskal test.

b χ^2^ test.

c Baseline serum levels of anti–glycoprotein Ib/IX antibody, anti–glycoprotein Ia/IIa antibody, and anti–glycoprotein IIb/IIIa antibody are unavailable in 7 subjects in the training cohort and all 59 subjects in the validation cohort.

d Baseline serum levels of anti–β2 glycoprotein I antibodies IgA, IgG, and IgM are unavailable in 26 subjects in the training cohort and all 59 subjects in the validation cohort.

### 3.4 Addition of drug(s) between day 6 and confirmed response in the training-cohort subjects

Compared with the subjects in the high-confidence fast response stratum, the subjects in the lower-confidence fast response and slow/no response strata received more types of drugs before reaching confirmed response. No high-confidence fast response subject received >3 types of ITP-targeting drugs (including glucocorticoid) between therapy start and confirmed response. In contrast, 2 (11%) of 18 lower-confidence fast response subjects and 7 (22%) of 32 slow/no response subjects received >3 types. The most common drug added between day 6 and confirmed response in the lower-confidence fast response and slow/no response strata was IVIG (*n* = 7), followed by danazol (*n* = 6), rhTPO (*n* = 5), eltrombopag (*n* = 2), vindesine (*n* = 2), and cyclosporine A (*n* = 1; Table 2).

### 3.5 Model validation in an independent cohort

There were 59 consecutive adults who were newly diagnosed with severe primary ITP and received glucocorticoid with or without ≥1 drug as initial therapy at the CCH. Baseline covariates of the validation cohort are displayed in Table 1. No subject received any ITP-targeting drug before admission to the CCH. The median baseline platelet count was 3 × 10^9^/L (IQR, 1-5 × 10^9^/L). Forty-four (75%), 25 (42%), and 13 (22%) subjects had ≥grade 3 cutaneous bleeding, ≥grade 2 mucosal bleeding, and ≥grade 2 internal bleeding, respectively. Glucocorticoids administered during days 1 to 5 included intravenous methylprednisolone (*n* = 49; median cumulative dose = 300 mg [IQR, 200-360 mg]), intravenous dexamethasone (*n* = 3; median = 25 mg [IQR, 25-37.5 mg]), and intravenous methylprednisolone plus dexamethasone (*n* = 7; methylprednisolone, median = 240 mg [IQR, 160-280 mg]; dexamethasone, median = 10 mg [IQR, 5-10 mg]). More than half (*n* = 33 [56%]) of the subjects in the validation cohort received ≥1 additional drug other than glucocorticoid(s) during days 1 to 5 (Table 1). The most common add-on medications were IVIG (*n* = 16 [48% of 33]), rhTPO (*n* = 14 [42%]), and “IVIG + rhTPO” (*n* = 3 [9%]).

Thirty-five (59%), 50 (85%), 52 (88%), and 52 (88%) subjects in the validation cohort achieved confirmed response by days 5, 10, 20, and 30, respectively. Concordance between the baseline platelet count and time to confirmed response was 0.53 (95% CI, 0.43, 0.62) and that between WHO bleeding score and time to confirmed response was 0.50 (95% CI, 0.47, 0.53).

**Figure 2:**
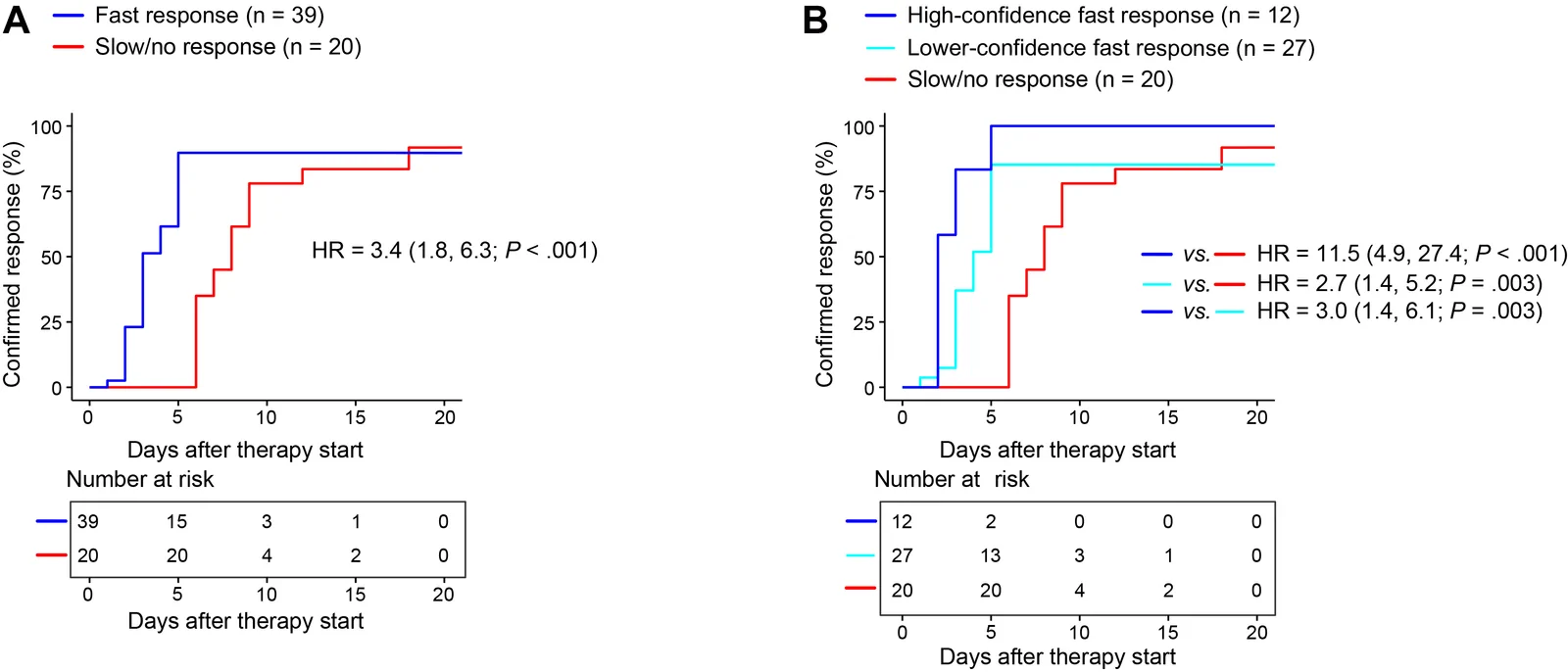
Cumulative incidence of confirmed response in the validation cohort. (A) ‘Fast response’ vs ‘slow/no response’. (B) ‘High-confidence fast response’ vs ‘lower-confidence fast response’ vs ‘slow/no response’.

Two (3%), 28 (47%), 27 (46%), and 2 (3%) validation-cohort subjects had 0, 1, 2, and ≥3 CBC measurements during days 1 to 5, respectively. Thirty-nine (66%) subjects were classified as fast response. The other 20 (34%) subjects, including 2 who did not undergo the CBC test during days 1 to 5, were classified as slow/no response. Concordance between fast response and a briefer interval to confirmed response was 0.71 (95% CI, 0.66, 0.77; Figure 2A). The daGOAT algorithm–trained model further classified the 39 fast-response subjects into high-confidence fast response (*n* = 12 [20%]) and lower-confidence fast response (*n* = 27 [46%]) (Figure 2B). Concordance between the daGOAT model–defined trinary classification and time to confirmed response was 0.78 (95% CI, 0.72, 0.83), significantly better than the concordance score of considering only the platelet count (*P* = .005). HR for confirmed response was 11.5 (95% CI, 4.9, 27.4; *P* < .001) and 2.7 (95% CI, 1.4, 5.2; *P* = .003) in the high- and lower-confidence fast response subjects, respectively, compared with the slow/no response subjects.

Age, bleeding scores, baseline values of platelet count and lymphocyte/monocyte ratio, and type of initial therapy were comparable between the 3 strata in the validation cohort (Table 2). Sex compositions of the 3 strata differed (*P* = .048). However, concordance between sex and time to confirmed response was only 0.51 (95% CI, 0.43, 0.59).

No high-confidence fast response subject in the validation cohort received ≥3 types of ITP-targeting drugs between therapy start and confirmed response. In comparison, 1 (4%) of 27 lower-confidence fast response subjects and 4 (20%) of 20 slow/no response subjects received ≥3 types of drugs. The most common add-on drug given between day 6 and confirmed response in the lower-confidence fast response and slow/no response subjects was rhTPO (*n* = 2 subjects), followed by IVIG (*n* = 1), eltrombopag (*n* = 1), and cyclosporine A (*n* = 1; Table 2).

### 3.6 Sensitivity analysis

Of the 144 subjects in our study cohort, 45 received only glucocorticoid(s) during days 1 to 5. Of these 45 subjects, 31 and 14 were fast response and slow/no response, respectively, and concordance between fast response and a briefer interval to confirmed response was 0.68 (95% CI, 0.61, 0.75). The daGOAT-trained model further classified the fast-response subjects into high-confidence fast response (*n* = 14) and lower-confidence fast response (*n* = 17). Concordance between the daGOAT model–defined trinary classification and time to confirmed response was 0.77 (95% CI, 0.70, 0.85) across the 45 subjects receiving only glucocorticoid(s) in initial therapy, significantly better than the concordance score of considering only the platelet count (*P* = .002).

### 3.7 Informativeness of individual covariates

**Figure 3:**
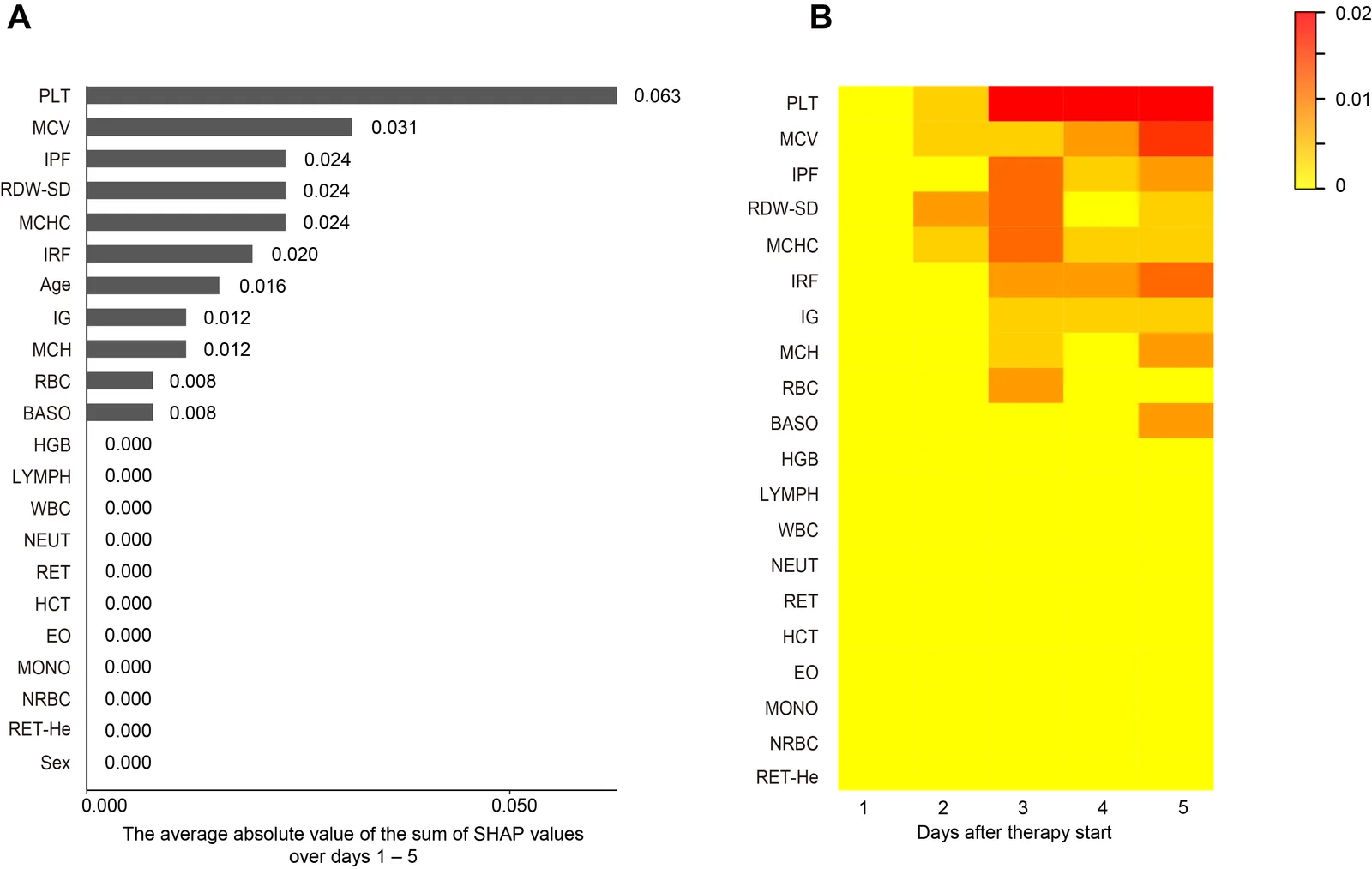
Informativeness of individual covariates for likelihood and speed of achieving confirmed response. (A) Relative overall importance of the covariates. (B) Relative daily contribution of the CBC covariates. BASO, basophil; EO, eosinophil; HCT, hematocrit; HGB, hemoglobin; IG, immature granulocyte; IPF, immature platelet fraction; IRF; immature reticulocyte fraction; LYMPH, lymphocyte; MCH, mean corpuscular hemoglobin; MCHC, mean corpuscular hemoglobin concentration; MCV, mean corpuscular volume; MONO, monocyte; NEUT, neutrophil; NRBC, nucleated red blood cell; PLT, platelet; RBC, red blood cell; RET, reticulocyte; RET-He, reticulocyte hemoglobin equivalent; RDW-SD, standard deviation of red cell distribution width; SHAP, SHapley Additive exPlanations; WBC, white blood cell.

SHAP analysis of the 85 subjects in the training cohort indicated that platelet count was the most informative CBC covariate, followed by MCV, IPF, RDW-SD, MCHC, and IRF (Figure 3A). Analysis of daily SHAP values indicated that, as a general rule, values of the CBC covariates during days 3 to 5 were more informative compared with values during days 1 to 2 (Figure 3B). Age was also among the most predictive covariates (Figure 3A).

A simplified model was fitted using the daGOAT algorithm based on response data and only 7 covariates, including platelet count, MCV, IPF, RDW-SD, MCHC, IRF, and age, from the training cohort. Concordance scores between model-computed subject classification and time to confirmed response were 0.78 (95% CI, 0.73, 0.83) and 0.79 (95% CI, 0.72, 0.86) in the training and validation cohorts, respectively, significantly better compared with considering only the platelet count (*P* < .001 and *P* = .001, respectively).

## 4 Discussion

Severe primary ITP is a rare, critical medical emergency that has not been thoroughly studied. A platelet count of ≤10 × 10^9^/L is associated with a high risk of critical bleeding including occult cerebral microbleeds and potentially life-threatening events [[24], [25], [26], [27]]. A primary objective of initial treatment in newly diagnosed severe primary ITP is rapid elevation of platelet count to mitigate this risk. However, complex pathophysiological mechanisms underlying ITP result in considerable interpatient variability in treatment response. Standard front-line glucocorticoid(s) might not elicit a prompt response in some patients with newly diagnosed severe primary ITP, who may require a higher dose of glucocorticoid(s) in initial treatment or early addition of nonglucocorticoid drugs. Thus, there is an urgent need for a tool that enables early prediction of confirmed response in patients with newly diagnosed severe primary ITP.

For newly diagnosed severe primary ITP, our data indicate that baseline platelet count and WHO bleeding score do not correlate with time to confirmed response. Therefore, there does not appear to be any simple rule based on baseline, single-time-point characteristics of patients that can be used to predict swifter confirmed response. We hypothesized that dynamic, multiple-time-point patterns of CBC data may reliably predict time to confirmed response in newly diagnosed severe primary ITP. Progress (or lack thereof) towards confirmed response is plausibly a complex physiological process that requires monitoring of a high number of hematological parameters having various levels of association with the likelihood of confirmed response. Prediction accuracy of our proposed model was replicated in 2 independent cohorts of patients. Because CBC is a common, convenient laboratory test, our model has the potential to be broadly applicable to adult patients with newly diagnosed severe primary ITP requiring rapid evaluation of treatment response. We have made the model publicly accessible as an online calculator for research validation purposes (https://itp-calculator.shinyapps.io/shiny_itp/). The online calculator upholds data security and privacy and does not perform statistics on or store any uploaded data or their associated meta-data. Alternatively, desensitized training data and computational code for training the model can be downloaded from https://github.com/chenjunren-ihcams/itp-calculator, and the model can be built locally on researchers’ private computers. We caution that the calculator is intended as a risk stratification aid, not a tool for treatment decision-making, and will need further validation.

SHAP analysis indicates that the most predictive covariates are age and the dynamic values of platelet count, MCV, IPF, RDW-SD, MCHC, and IRF. IPF is a well-known indicator of elevated, compensating thrombopoietic activity in ITP, and its value declines when the patient is responsive to therapy [[28], [29], [30]]. Alterations in red blood cell covariates may reflect changes in the disease status of newly diagnosed severe primary ITP. Red blood cell–related covariates including MCHC and RDW-SD have been reported to correlate with severity of inflammation in various diseases [[31], [32], [33]]. Alternatively, observed changes in the CBC covariates might reflect bone marrow compensation in response to bleeding-related erythropoietic stress or treatment-related hematologic dynamics.

In a recent prospective study using a daGOAT algorithm–trained AI model, it was demonstrated that proactive identification of patients at risk for severe acute graft-*versus*-host disease may guide pre-emptive pharmaceutical intervention in adults who have received human leukocyte antigen-haploidentical hematopoietic cell transplantation [21]. We submit that, likewise, rapid evaluation of response likelihood and response speed in newly diagnosed severe primary ITP using our proposed calculator may assist decision on timely therapy adjustment to accelerate recovery of the platelet count [34,35]. Data from our study subjects suggest that lower-confidence fast responders and slow/no-responders may require more proactive interventions. Continuing treatment that is limited to glucocorticoid and/or IVIG for 6 weeks, as suggested in some of the current clinical practice guidelines [3,4], might delay response in these patients and prolong their hospitalization. We hypothesize that lower-confidence fast responders and slow/no-responders may benefit from early addition of TPO-RA and/or newer modalities of pharmaceutical intervention, thereby bypassing lengthy, potentially futile treatment when their conditions remain critical. This hypothesis will need to be prospectively tested in a randomized controlled trial. We propose that lower-confidence fast responders and slow/no-responders are potential candidates for enrollment in prospective trials of new treatment strategies.

Our study has limitations. First, retrospective analyses are subject to potential attendant biases, and prospective clinical utility testing has not been conducted. Second, our sample size was limited. Although having a validation cohort is a strength of our study, there remains the risk of overfitting, and we acknowledge the need for larger-scale validation using data from multiple centers. Third, other centers might conduct the CBC test less frequently after initiation of therapy compared with our centers. Ninety-seven percent of our study cohort underwent at least 1 CBC test during days 1 to 5. Fourth, generalizability to other centers with different front-line treatment strategies might be limited. Model performance in non-Chinese cohorts and cohorts with different age distributions or alternative treatment paradigms remains unknown. Fifth, we did not study immune cell sub-populations because they were not serially monitored in the patients in our study cohorts. Nor did we study other candidate biomarkers such as variables along the gut-immune axis, cytokines (eg, TGF-β1 and IL10-to-IL17 ratio), and complement activation profiles that have received increasing attention for their potential value in the prognosis of ITP [[36], [37], [38], [39], [40]]. We anticipate that AI-assisted prediction of treatment response in newly diagnosed adult severe primary ITP will benefit from incorporation of a broader range of clinical covariates.

Our study should be treated as hypothesis-generating. Our tested AI model and the AI-driven calculator that is made publicly accessible, through early prediction of likelihood and speed of reaching confirmed response in adults with newly diagnosed severe primary ITP, might assist timely adjustment of therapy to improve response. However, this hypothesis needs to be tested in prospective clinical trials. Validation in non-Chinese cohorts and cohorts where initial therapy with TPO-RA and/or other newer drug(s) is more common is also needed.
